# Comparative Characterization of Protein Hydrolysates from Three Edible Insects: Mealworm Larvae, Adult Crickets, and Silkworm Pupae

**DOI:** 10.3390/foods8110563

**Published:** 2019-11-09

**Authors:** Sungwon Yoon, Nathan A. K. Wong, Minki Chae, Joong-Hyuck Auh

**Affiliations:** Department of Food Science and Technology, Chung-Ang University, Anseong 17546, Korea; ysw0812@naver.com (S.Y.); nathanwong52@gmail.com (N.A.K.W.); alsrl3433@naver.com (M.C.)

**Keywords:** protein hydrolysates, mealworm, cricket, silkworm pupae

## Abstract

A comparative characterization of proteins from three edible insects—*Tenebrio molitor* (mealworm) larvae, *Gryllus bimaculatus* (cricket), and *Bombyx mori* (silkworm) pupae—was performed in this study. Proteins were extracted from edible insects and their hydrolysates were prepared through enzymatic hydrolysis with commercial enzymes (Flavourzyme: 12%; Alcalase: 3%). Solubility was significantly higher following enzymatic hydrolysis, while foamability was lower compared to those of the protein control. Angiotensin-converting enzyme was significantly inhibited after enzymatic hydrolysis, especially following Alcalase treatment, with IC_50_ values of 0.047, 0.066, and 0.065 mg/mL for *G. bimaculatus*, *T. molitor* larvae, and *B. mori* pupae, respectively. Moreover, the Alcalase-treated group of *B. mori* pupae and the *T. molitor* larvae group treated with a mixture of enzymes showed the effective inhibition of α-glucosidase activity. The anti-inflammatory activity of the insect hydrolysates was assessed via nitric oxide production from macrophages, and *B. mori* pupae samples exhibited significant activity regardless of the method of hydrolysis. These results indicate the functional properties of protein and hydrolysates from three species of edible insects, which may be useful in their future exploitation.

## 1. Introduction

In the food industry, proteins are being used as integral sources not only because of their nutritive value but also because of their functional properties [1]. Some of the required functional properties of food proteins for application in food formulations include their solubility, gelation ability, emulsifying activity, foaming capacity, and biological activity [2,3]. Food protein and its hydrolysates have been used in diverse applications including beverages, food ingredients, cosmetics, and functional foods [4].

The demand for food protein is expected to double by 2050 due to the expected increase in the global population [5]. This population will require a supply of protein from innovative and sustainable sources [6], and insects have been recognized as among the novel alternative sources that could fulfill protein demands in the future [7]. Ninety-five percent of the animal kingdom consists of insects, which as a group, are extremely biodiverse [8]. There are several advantages to using insects as compared to the existing protein sources, such as fewer greenhouse gas emissions, less water and land used, and a higher efficiency of protein conversion [5]. Additionally, insect protein has a good amino acid profile, with essential amino acids (i.e., Leu, Ile, Lys, Met, Thr, Trp, Phe, and Val) being found in a diverse set of insect species [5]. Thus, insects are receiving attention as a good source of protein with various functional properties. Their functionality can also be enhanced or controlled by enzymatic hydrolysis [2,4].

Interestingly, consuming insects, or entomophagy, is already being practiced worldwide by 2 billion people in nearly 113 counties and has been practiced for centuries [9]. For instance, approximately 1900 species of insects have been used as food in Africa, Asia, and Latin America [5]. Recently, Govorushko reviewed the global status of edible insects and pointed out that insects for food use is a promising direction in the future [10]. In South Korea, seven species of insects, including two-spotted crickets (*Gryllus bimaculatus*), mealworm (*Tenebrio molitor*) larvae, silkworm (*Bombyx mori*) pupae, dynastid beetle larvae (*Allomyrina dichotoma*), white-spotted flower chafer larvae (*Protaetia brevitarsis seulensis*), field grasshopper (*Sphenarium purpurascens*), and *Bombyx batryticatus*, are currently recognized as edible insects [11].

Although the consumption of insects has many advantages, low consumer acceptance in developed countries that typically do not eat insects remains a barrier to its widespread adoption [12,13]. In these cases, perhaps the best method for capitalizing on the advantages of entomophagy is to utilize insect proteins as food ingredients depending on their physicochemical properties [14] such as solubility, emulsifying/foam stability, or gel forming activity. Many of these properties can be controlled by enzymatic modification [9]. For example, pretreated (defatted and acid-hydrolyzed) mealworm larvae (*T. molitor)* and silkworm pupae (*B. mori*) were investigated as novel ingredients in emulsion sausages with no adverse effects [6]. Furthermore, proteins from grasshoppers (*Schistocerca gregaria*) and honey bees (*Apis mellifera*) were also demonstrated to have foaming/emulsifying properties that were comparable to the properties of proteins from mealworm larvae and silkworm pupae, which suggests that they could also be used as an additive [15]. More recently, Mintah et al. [16] demonstrated that ultrasonic processing contributes significant changes in various functional attributes of edible insect proteins and hydrolysates. In terms of the physiological activities of insect proteins from edible species, several insects have been shown to inhibit the angiotensin-converting enzyme (ACE) [17], while peptides from silkworm pupae (*B. mori*) displayed α-glucosidase inhibitory activity [18]. Moreover, silkworm hemolymph was previously observed to have anti-inflammatory properties [19]. Similarly, protein from crickets (*G. sigillatus),* mealworm (*T. molitor*) larvae, and desert locusts (*S. gregaria)* have also shown anti-inflammatory effects after protein hydrolysis and peptide fractionation [20].

Although the properties of some insect proteins and hydrolysates have been identified, the number of studies is still quite limited. In the present study, we performed a comparative characterization of insect proteins and their hydrolysates from three major edible insects (mealworm larvae, crickets, and silkworm pupae) permitted in Korea, and their basic physicochemical and physiological properties were evaluated.

## 2. Materials and Methods

### 2.1. Materials and Reagents

Three insects that are recognized as edible in Korea were used in this study. Specifically, adult two-spotted crickets (*G. bimaculatus*), mealworm (*T. molitor*) larvae, and silkworm (*B. mori*) pupae were obtained from Insect Vision (Yangju, Korea). All insects arrived dried and, to preserve these specimens, they were immediately stored at −50 °C until use. Unless specified, all reagents utilized were of chemical grade and obtained from the following suppliers: Bio-Rad^®^ (Hercules, CA, USA), Sigma-Aldrich^®^ Corp. (St. Louis, MO, USA), and Daejung Chemicals & Metals Co. (Siheung, Seoul, South Korea). Methanol and *n*-hexane were purchased from Honeywell Burdick & Jackson^®^ (Thomas Scientific, Swedesboro, NJ, USA). The enzymes including Flavourzyme 1000 L, Alcalase 2.4 L, Neutrase, and Protamex were provided by Novozymes (Bagsvaerd, Denmark). Bovine serum albumin powder was purchased from Equitech-Bio, Inc. (Kerrville, TX, USA). Additional materials, including sodium carbonate, glycerol, and tetramethyl-ethylenediamine (TEMED), were obtained from Duksan Pure Chemicals Inc. (Seoul, South Korea), Samchun Chemical Co., Ltd. (Seoul, South Korea), and Taeshin Bio-Science (Seoul, South Korea), respectively.

### 2.2. Defatting and Protein Extraction

Lipids were removed and proteins extracted from insects via a method outlined previously [21]. The insect samples were first defatted using food-grade *n*-hexane to increase the protein yield. A 1:20 ratio of *n*-hexane (*w*/*v*) was applied and stirred for 36 h with filtering and replacing the hexane at 12 h intervals. The defatted sample was left to dry overnight under a fume hood at room temperature. Following the hexane defatting, proteins were extracted from these insects by sonication in a Sonics^®^ Vibra-Cell™ VCX 750 ultrasonic unit (Sonics & Materials Inc., Newtown, CT, USA). The sonicator was set to 20 kHz for 15 min, with a 75% AMPL (amplitude) and pulsed every 3 s with a 1 s interval. Protein extracts were filtered, lyophilized, and then stored at −50 °C until further use.

### 2.3. Preparing Protein Hydrolysates

The freeze-dried extracted samples were rehydrated with ultrapure water and treated with four commercial proteases. Two doses (Flavourzyme with 30 and 60 U/g protein; Alcalase with 12 and 72 mU/g protein; Neutrase with 4 and 24 mU/g protein; Protamex with 7.5 and 45 mU/g protein) were applied in order to obtain effective protein hydrolysis. In addition to these four treatment groups, a cotreatment group (mixture group) was subjected to both Flavourzyme and Alcalase (Flavourzyme 30 U with Alcalase 12 mU/g protein; Flavourzyme 60 U with Alcalase 72 mU/g protein). Samples were treated with these proteases at 55 °C for 8 h. Aliquots of each sample were taken at 2 h intervals to assess the molecular weight distribution and degree of the hydrolysis of the insect proteins. The crude hydrolysates obtained after enzyme treatment were centrifuged and the supernatants were freeze-dried for further assessment.

### 2.4. SDS-PAGE

The molecular weight distribution of the insect proteins was determined by SDS-PAGE using a 15% polyacrylamide resolving gel. The protein concentration was determined by Bradford assay. Electrophoresis was performed at 140 V followed by staining and destaining. The gel was then visualized using a Molecular Imager^®^ Gel Doc™ XR+ (Bio-Rad, Hercules, CA, USA).

### 2.5. Solubility (%)

The solubility of the hydrolysates was determined according to the method by Pacheco-Aguilar et al. [22] through a dry-weight comparison with some modifications. After enzymatic hydrolysis, the hydrolysates were centrifuged (10,000× *g*, at 4 °C for 15 min) to obtain the soluble protein fraction (supernatant), which was freeze-dried prior to measurement. Solubility (%) was calculated using the total weight of the sample and the soluble portions with the following equation:

Solubility (%) = (Weight of soluble proteins/Weight of total protein) × 100 (%)(1)

### 2.6. Emulsifying Activity and Stability

The emulsifying properties (the activity emulsifying index (EAI) and the emulsion stability index (ESI)) were determined using a spectrophotometric method according to Hall et al. [9] with some modifications. Hydrolysates (0.5% (*w*/*v*)) in distilled water were mixed with pure soybean oil at the ratio 3:1 and then sonicated for 2 min (3 s pulse per 6 s rest, 30% AMPL). Aliquots were immediately diluted in 0.3% SDS solution (200 fold dilution) and the absorbance at 500 nm was measured. Then, the emulsifying activity index (EAI) was calculated as follows:

(2) $$EAI=\frac{\left(2T\right)\times \left(A\right)\times \left(df\right)}{\left(\emptyset \right)\times \left(c\right)\times \left(10,000\right)}$$

where *T* is the turbidity of the solution at 500 nm at time zero, *A* is the absorbance of the solution, *df* is the dilution factor, *ø* is the volume of the oil fraction (0.25), and *C* is the concentration of protein (0.5 g/mL).

The emulsion stability index (ESI) was calculated by measuring the aliquots of the emulsions taken at different time intervals (20, 40, and 60 min) based on the absorbance at 500 nm. The ESI was calculated as follows:

(3) $$\%\mathrm{ESI}=100-\left[\frac{EAI_{0}-EAI_{T}}{EAI_{0}}\right]\times 100$$

where *EAI*_0_ is the measurement of *EAI* at time zero (0 min) and *EAI_T_* is the *EAI* at the time interval tested (20, 40, and 60 min).

### 2.7. α-Glucosidase Inhibitory Activity

The α-glucosidase inhibitory activity of the hydrolysates was measured according to Kwon et al. [23] with some modifications. α-glucosidase (0.7 U/mL) and 5 mM of *p*-nitrophenyl-α-d-glucopyranoside (*p*-NPG) (Sigma Co.) was dissolved in a 50 mM phosphate buffer (pH 7.0) and used as an enzyme solution and a substrate solution, respectively. Forty microliters of the α-glucosidase enzyme was added to each sample solution (240 µL of 10 mg/mL) in a 96-well plate and pre-incubated at 37 °C for 5 min. Subsequently, 40 µL of *p*-NPG substrate was added and the absorbance was measured at 405 nm in kinetic mode for 20 min at 37 °C. The inhibitory activity was calculated as a percentage of the blank control.

### 2.8. ACE Inhibitory Activity

An in vitro assay to measure the ACE inhibitory activity of the hydrolysates was performed as described by Chang et al. [24] with some modifications. One hundred microliters of the hydrolysate samples, 100 µL of ACE (40 mU/mL), and 100 µL of 15 mM hippuryl-l-histidyl-l-leucine (HHL; as a substrate dissolved in a 20 mM sodium borate buffer with 0.3 M NaCl (pH 8.3)) were incubated in a 96-well plate for 2 h at 37 °C. The enzymatic reaction was terminated by adding OPA reagent (*o*-phthaladelhyde), and the absorbance was measured at 390 nm after incubation at 25 °C for 20 min. The inhibitory activity (%) was calculated as follows:

(4) $$\mathrm{Inhibition}\;(\%)=\left[1-\frac{A1-A2}{A3-A4}\right]\times 100$$

where A1 is the absorbance of the mixture of hydrolysate samples, HHL, and ACE; A2 is the absorbance of the mixture of samples and buffer; A3 is the absorbance of the mixture of HHL, ACE, and buffer; and A4 is the absorbance of only the buffer.

### 2.9. Cell Proliferation Assay (3-(4,5-dimethylthiazol-2-yl)-2,5-diphenyltetrazolium Bromide (MTT) Assay)

RAW 264.7 cells were obtained from American Type Culture Collection (ATCC, Rockville, MD, USA) and cultured in Dulbecco’s modified eagle’s medium (DMEM) supplemented with 10% fetal bovine serum (FBS), 1% penicillin–streptomycin, and 1% HEPES at 37 °C and 5% CO_2_. For subculture, RAW 264.7 cells were rinsed with Dulbecco’s phosphate buffered saline (DPBS), mechanically scraped from the plates, and plated in 10 cm culture dishes. Cell proliferation was investigated using the MTT assay. RAW 264.7 cells were plated in a 96-well plate (4 × 10^4^ cells/well) and incubated overnight. Plated cells were treated with 100–500 μg/mL of samples and incubated for 20 h. MTT (5 mg/mL, 20 μL) was added to the cell suspensions and incubated for 4 h. After aspirating the medium from the wells, 200 μL of DMSO was added to dissolve the formazan crystals. The absorbance was measured using a microplate reader (Molecular devices, Sunnyvale, CA, USA) at 570 nm. Cell proliferation was described as the relative cell viability compared to that of the control cells (medium alone, without sample treatment).

### 2.10. Measurement of Anti-Inflammatory Activity (Nitric Oxide (No) Assay)

RAW 264.7 cells were plated in a 96-well plate (4 × 10^4^ cells/well) and incubated overnight at 37 °C and 5% CO_2_. Plated cells were treated with 100 ng/mL lipopolysaccharide (LPS) and these samples for 20 h, and NO production from RAW 264.7 cells was then quantified. Specifically, after 20 h, the supernatants (50 μL) were mixed with 50 μL of sulfanilamide solution (1% sulfanilamide in 5% phosphoric acid) for 10 min at room temperature. Fifty microliters of 0.1% N-1-napthylethylenediamine dihydrochloride in water was then mixed into that solution for 10 min at room temperature. The absorbance at 540 nm was measured using a microplate reader (Molecular devices), and NO concentration was determined based on the standard curve with sodium nitrite.

### 2.11. Statistical Analysis

All trials were performed in triplicate unless indicated otherwise. Data was analyzed using SPSS^®^ ver.25 (IBM, Armonk, NY, USA). Statistical analyses of observed differences among means consisted of analysis of variance (ANOVA) using Duncan’s multiple-range test with a level of significance defined at 5%.

## 3. Results

### 3.1. Hydrolysis Pattern of Insect Proteins

**Figure 1:**
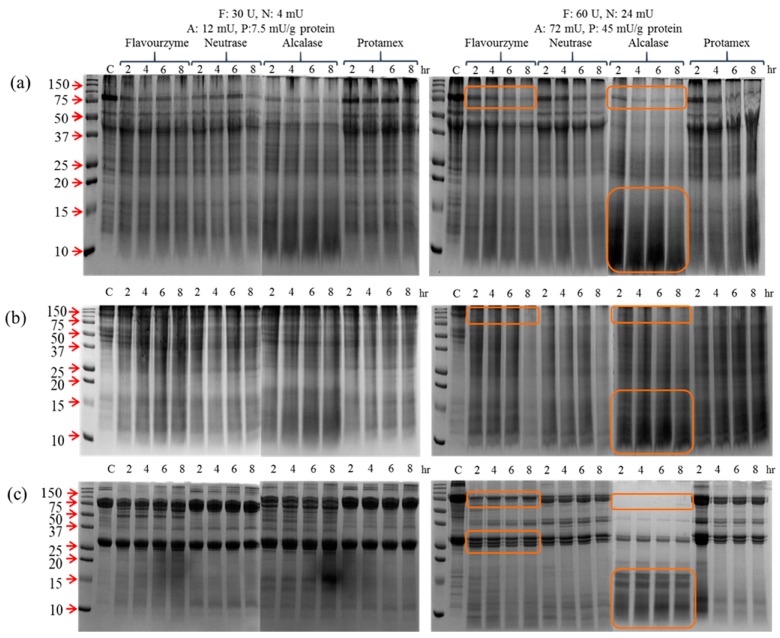
Hydrolysis pattern of (**a**) mealworm, (**b**) cricket, and (**c**) silkworm pupae protein extracts by four commercial proteases. Two different doses of enzymes were applied. Left: Flavourzyme: 30 U, Neutrase: 4 mU, Alcalase: 12 mU, and Protamex: 7.5 mU/g protein. Right: Flavourzyme: 60 U, Neutrase: 24 mU, Alcalase: 72 mU, and Protamex: 45 mU/g protein. The numbers above the lanes signify the treatment times in hours.

The hydrolysis pattern of insect proteins was investigated using SDS-PAGE, as indicated in Figure 1. Four kinds of commercial proteases (food grade) were applied to the insect samples at two doses, and as a result, the dramatic hydrolysis of insect proteins was observed in all samples. Flavourzyme- and Alcalase-treated groups displayed a relatively higher hydrolyzing activity with a reduction in a 75 kDa band to a band below 15 kDa post-hydrolysis. With respect to mealworm larvae, the thick band at 75 kDa became thinner and even a small protein band below 15 kDa was produced by Alcalase treatment (Figure 1a). The main bands from the cricket proteins at 150 and 50 kDa were gradually hydrolyzed to bands below 15 kDa after enzyme treatment (Figure 1b). The silkworm pupae proteins showed the clearest pattern of hydrolysis as compared to the others (Figure 1c), with major proteins at 75 and 25 kDa being degraded to proteins less than 15 kDa in size. The Alcalase-treated group showed the highest degree of hydrolysis among the samples. While treatment was performed for up to 8 h, no significant changes were observed in the hydrolysis pattern after 4 h. Flavourzyme and Alcalase showed the highest hydrolysis efficiencies, and thus, they were used to treat samples in the subsequent experiments.

**Figure 2:**
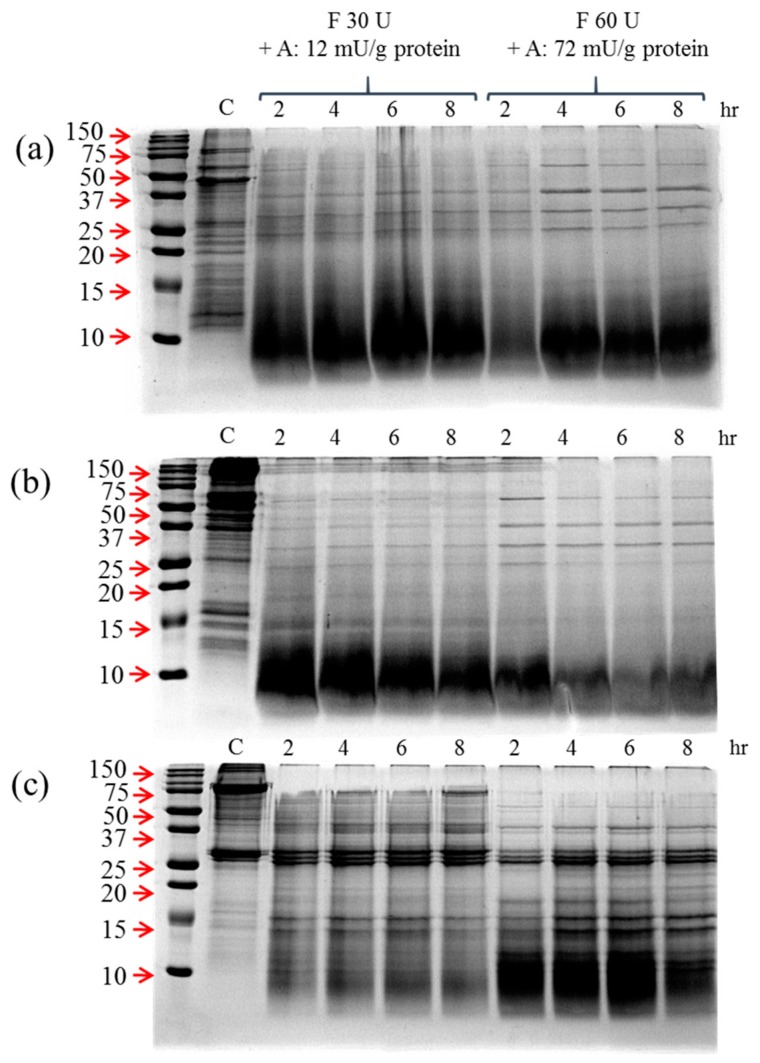
Hydrolysis pattern of (**a**) mealworm, (**b**) cricket, and (**c**) silkworm pupae protein extracts through using Flavourzyme and Alcalase enzyme mixtures. Two different doses of enzymes were applied. Left: Flavourzyme: 30 U + Alcalase: 12 mU/g protein. Right: Flavourzyme: 60 U + Alcalase: 72 mU/g protein. The numbers above the lanes signify the treatment times in hours.

We also investigated the effect of Flavourzyme and Alcalase cotreatment on the hydrolysis patterns of insect proteins (Figure 2). Two different doses of enzyme mixture were applied, and the hydrolysis patterns were investigated with SDS-PAGE analysis. In all cases, a significantly higher ratio of small proteins was observed for these samples when compared to the hydrolysis patterns in Figure 1. The cotreatment seemed to exert a synergistic effect for the hydrolysis insect proteins.

### 3.2. Solubility

**Figure 3:**
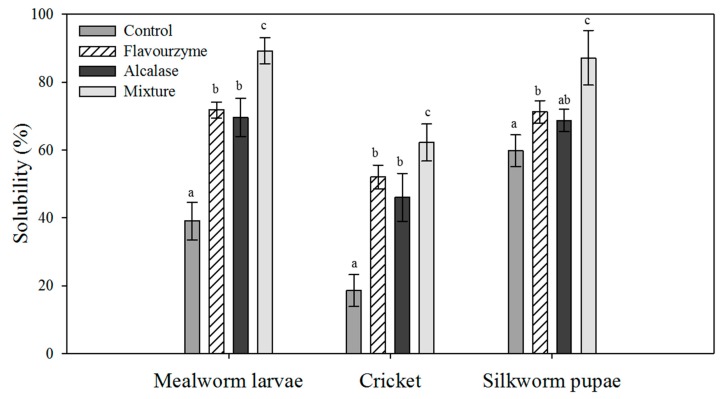
The solubility of insect proteins before and after enzymatic hydrolysis. Each value is expressed as the mean ± standard deviation (SD) (*n* = 3). Values marked with different letters indicate significant differences among treated groups (*p* < 0.05).

The solubility of insect proteins varied, with cricket proteins having the lowest solubility among the tested samples. Enzymatic hydrolysis significantly increased protein solubility in all samples while cotreatment with two enzymes (Flavourzyme 60 U with Alcalase 72 mU/g protein) exhibited the highest solubility in all insect species (Figure 3). Cricket proteins showed a relatively lower solubility (18.5%–62.2%), whereas a dramatic increase in solubility was observed in mealworm larvae. Hydrolysates of mealworm larvae showed a higher solubility (69.6%–89.2%) than the native, unhydrolyzed protein (39.1%).

### 3.3. Emulsifying Activity and Stability

**Figure 4:**
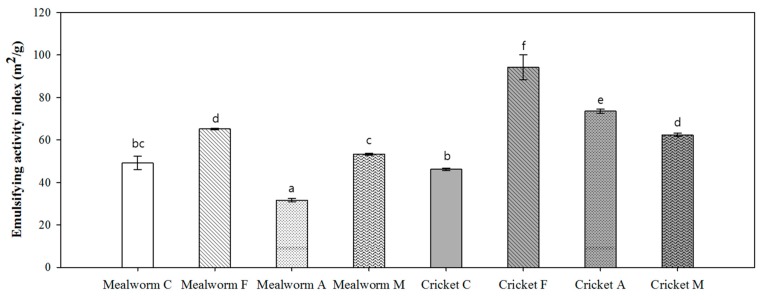
The emulsifying activity index (EAI) of insect proteins and their hydrolysates. The results are reported as m^2^/g. Each value is expressed as the mean ± SD (*n* = 3); values marked with different letters indicate significant differences among treated groups (*p* < 0.05). C, control; F, Flavourzyme; A, Alcalase; M, Flavourzyme + Alcalase.

**Figure 5:**
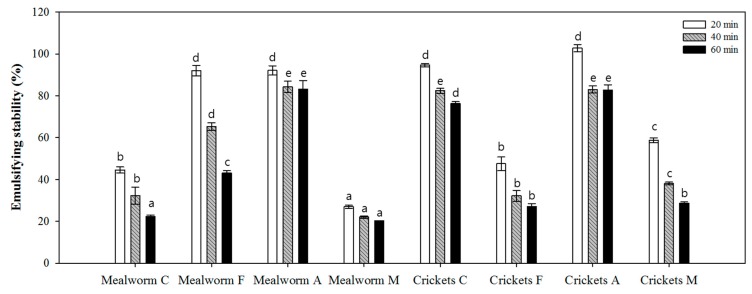
The emulsifying stability index (ESI) of insect proteins and their hydrolysates after 20, 40, and 60 min of emulsion formation. Results are reported as % ESI. Each value is expressed as the mean ± SD (*n* = 3); values marked with different letters indicate significant differences among treated groups (*p* < 0.05). C, control; F, Flavourzyme; A, Alcalase; M, Flavourzyme + Alcalase.

Among the basic properties of food ingredients, the emulsifying activity and stability of insect proteins was measured and compared in Figure 4 and Figure 5. In this experiment, aggregates were formed when silkworm pupae proteins/hydrolysates were used; thus, they did not possess any detectable emulsifying activity. Generally, the emulsifying activity increased significantly when samples were treated with proteases, except for Alcalase-treated mealworm proteins (Figure 4). Among the hydrolysates, the Flavourzyme-treated samples showed the greatest emulsion capacities. The highest emulsifying activity (94.3 m^2^/g) was determined for Flavourzyme hydrolysates of cricket proteins. However, that hydrolysate showed lower emulsifying stability of 47.5%, 32.2%, and 27.1% after 20 min, 40 min, and 60 min, respectively (Figure 5). Cotreated hydrolysates (Flavourzyme 60 U with Alcalase 72 mU/g protein) displayed the lowest stability out of all treatment groups within the same species. In addition, Alcalase-treated samples showed significantly higher stability than other hydrolysates. Given both properties, cricket hydrolysates treated with Alcalase displayed higher emulsifying activity with stability.

### 3.4. ACE Inhibitory Activity

**Figure 6:**
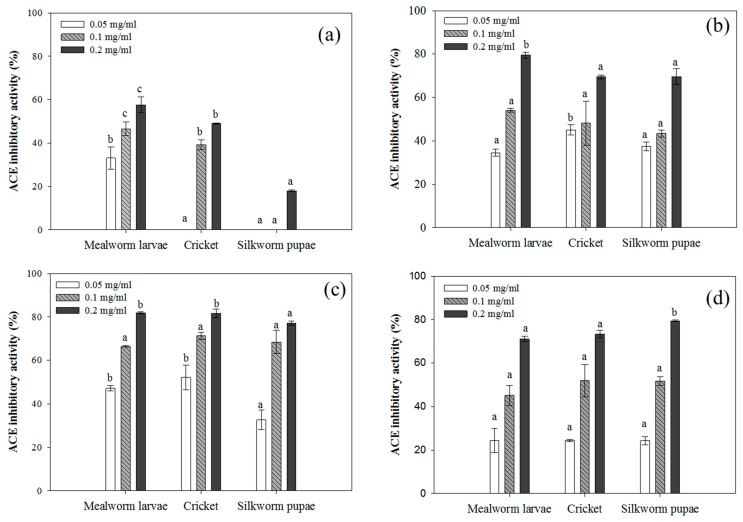
Angiotensin-converting enzyme (ACE) inhibitory activity of (**a**) insect proteins and their hydrolysates following (**b**) Flavourzyme, (**c**) Alcalase, and (**d**) mixture (Flavourzyme + Alcalase) treatments. Each value is expressed as the mean ± SD (*n* = 3); values marked with different letters indicate significant differences among treated groups (*p* < 0.05).

The ACE inhibitory activity of insect hydrolysates is summarized in Figure 6. Overall, ACE inhibition significantly increased following the enzymatic hydrolysis of insect proteins, especially after the Alcalase treatment. Hydrolysates of silkworm pupae displayed the most dramatic increase in activity (43 to 68%) at 0.1 mg/mL as compared to untreated protein samples that were estimated to have no inhibitory activity. With respect to unhydrolyzed protein samples, mealworm larvae displayed the highest level of ACE inhibition. In all cases, Alcalase-treated samples showed the greatest ACE inhibitory effect when compared to the other treatments. Most of all, Alcalase-hydrolyzed cricket proteins were the most effective at inhibiting ACE, with an IC_50_ value of 0.047 mg/mL.

### 3.5. α-Glucosidase Inhibitory Activity

**Figure 7:**
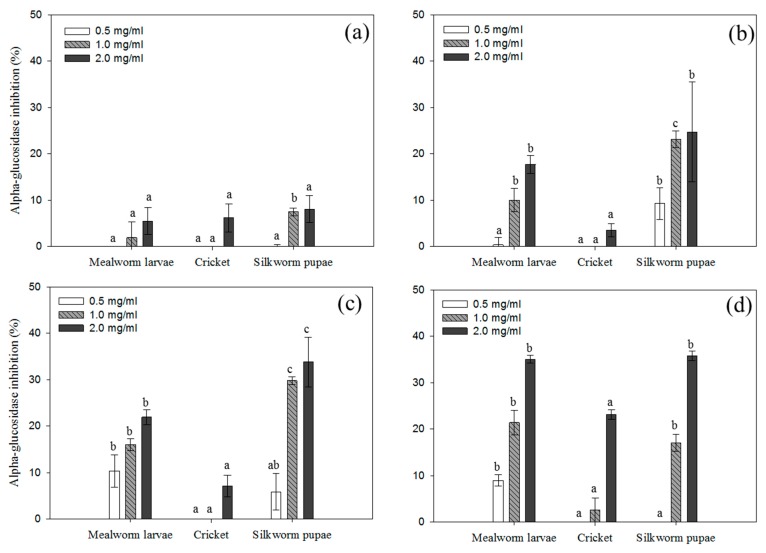
α-Glucosidase inhibitory activity of (**a**) insect proteins and their hydrolysates following treatment with (**b**) Flavourzyme, (**c**) Alcalase, and (**d**) a mixture of Flavourzyme and Alcalase. Each value is expressed as the mean ± SD (*n* = 3); different letters indicate significant differences among treated groups (*p* < 0.05).

The in vitro α-glucosidase inhibitory activity of insect proteins and their hydrolysates was assessed to estimate the anti-diabetic activity of these isolates (Figure 7). The inhibitory activities were significantly enhanced following the enzymatic hydrolysis of the insect proteins in all treated groups in a dose-dependent manner. Untreated insect proteins did not exert any notable inhibitory activity on α-glucosidase activity, with less than 10% inhibition occurring even at high concentrations of protein (2 mg/mL; Figure 7a). Among the hydrolysates, Alcalase-treated silkworm pupae and Flavourzyme + Alcalase-treated mealworm larvae exhibited significant inhibitory activity on α-glucosidase.

### 3.6. Anti-Inflammatory Activity

**Figure 8:**
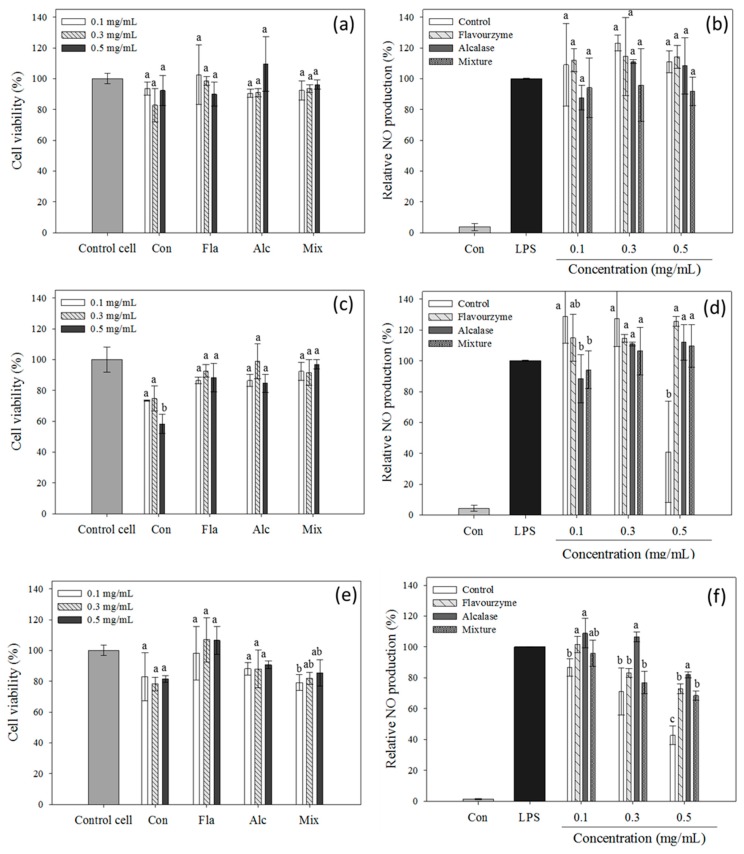
Cell viability ((**a**), (**c**), (**e**)) and nitric oxide (NO) production ((**b**), (**d**), (**f**)) from lipopolysaccharide (LPS)-induced RAW264.7 cells. Cells were treated with 100 ng/mL LPS and protein samples (unhydrolyzed or hydrolyzed insect proteins) and then NO production was measured. (**a**) effect on cell viability by mealworm larvae, (**b**) effect on NO production by mealworm larvae, (**c**) effect on cell viability by cricket, (**d**) effect on NO production by cricket; (**e**) effect on cell viability by silkworm pupae, (**f**) effect on NO production by silkworm pupae. Each value is expressed as the mean ± SD (*n* = 3); different letters indicate significant differences among treated groups (*p* < 0.05).

The cytotoxicity of the insect proteins and their hydrolysates was evaluated before determining their anti-inflammatory activity. Cytotoxicity was measured using the MTT assay, and no cytotoxicity was observed on RAW 264.7 cells at the tested protein concentrations (0.1, 0.3, and 0.5 mg/mL) except for the cricket proteins, which lowered the cell viability to less than 80% as compared to the control (Figure 8c). LPS-induced NO production in RAW 264.7 cells was assessed as an indicator of anti-inflammatory activity (Figure 8b,d,f). Mealworm larvae proteins and their hydrolysates did not show anti-inflammatory activity (Figure 8b). The reduced level of NO production by cricket protein is a reflection of the cytotoxicity of the proteins themselves. Moreover, the hydrolysates did not exhibit anti-inflammatory activity either (Figure 8d). Silkworm pupae proteins and their hydrolysates significantly reduced NO production. Over 50% of the NO production was reduced with 0.5 mg/mL of silkworm protein, and Flavourzyme-treated hydrolysates also exerted a significant reduction in NO level.

## 4. Discussion

In recent years, peptides and proteins have become common food ingredients and nutraceuticals, given their physiochemical and physiological properties [1,4]. However, as the demand for protein increases, it is going to be increasingly important to find new protein sources. That said, insects are garnering significant interest as a protein source because they are sustainable, eco-friendly, and nutritious [5]. However, consuming insects is still unfamiliar in many countries due to food neophobia [12,13]. Recent consumer studies suggest that introducing “invisible insects” into food may enhance consumer awareness and make the idea more palatable [7]. The production and application of protein hydrolysates are among the most promising ways of overcoming consumer fear regarding eating insects, and different types of commercial enzymes have been used to prepare insect protein hydrolysates [8,9,25,26]. In this study, four commercial enzymes (Flavourzyme, Neutrase, Alcalase, and Protamex) were applied to prepare protein hydrolysates, and Flavourzyme and Alcalase were selected as the most efficient in hydrolyzing insect proteins. These two enzymes cleave proteins in opposite ways, with Flavourzyme being an exopeptidase and Alcalase being an endopeptidase. SDS-PAGE analysis revealed that enzymatic hydrolysis for 4 h was sufficient to efficiently hydrolyze the target proteins. This is in line with a previous study that showed that silkworm pupae protein was sufficiently hydrolyzed using alkaline protease after a 3 h treatment [27].

The solubility of a protein or protein mixture is an important characteristic when considering their use as a food ingredient [9]. Typically, protein hydrolysates are more soluble than unhydrolyzed proteins resulting in a reduction in protein molecular weight and an increase in protein hydrophilicity [28,29]. In this study, enzymatic hydrolysis reduced protein size and increased protein solubility. Moreover, cotreatment with Alcalase and Flavourzyme displayed the highest solubility for insect proteins among the treatment groups. Improved solubility is the most notable effect of protein hydrolysis on protein properties, but the hydrolysis process needs to be controlled since unfavorable effects, such as a bitter taste, can occur [29,30].

The solubility of enzymatic hydrolysates can also be affected by pH. For instance, protein hydrolysates of *T. molitor* larvae were previously evaluated through a pH range of 2 to 10, following treatment with an alkaline protease [31]. In that study, the solubility increased at higher pH, where it was also affected by the existence of ions. The solubility of adult tropical band cricket hydrolysates (*G. sigillatus)* obtained through Alcalase treatment confirmed these observations, with increased protein solubility being observed at higher pH values [9]. In the present study, we ran the enzymatic reactions in distilled water for the ease of its practical application. However, further optimization of the hydrolytic process may need to be performed before these hydrolysates are applied as a food additive.

Proteins serve as good emulsifiers due to their amphipathic characteristics and their ability to unfold and reorganize their adsorption at the oil–water interface [28,29]. Emulsifying activity increases when the number of dispersed oil droplets decreases, and the adsorption ability of proteins or peptides affects the oil–water interface [22]. In this study, the emulsifying activity of insect proteins following enzymatic hydrolysis increased significantly when compared to unhydrolyzed proteins (mealworm larvae: 4.2–16.0 m^2^/g; crickets: 16.2–48.1 m^2^/g), except when Alcalase was used to treat mealworm proteins. The proteolytic process facilitates emulsion formation by exposing buried hydrophobic amino acid groups and releasing hydrophobic, surface-stabilizing residues [9]. Flavourzyme-treated samples that showed the highest EAI values (Figure 4) demonstrated that buried amino acid groups might be exposed by Flavourzyme-mediated hydrolysis, forming new interactions at the oil–water interface [28]. However, samples that were cotreated with the Flavourzyme and Alcalase enzyme mixture displayed lower EAI and ESI values among the hydrolysates. Jung et al. [32] demonstrated that some hydrolysis conditions can lead to a decrease or no alteration in the emulsifying capacity of hydrolysates, and extensive hydrolysis may cause a rapid loss in their emulsifying ability [22]. The emulsifying properties of insect proteins and their hydrolysates have been evaluated previously. For instance, Kim et al. [6] suggested that pretreated mealworm larvae and silkworm pupae flour could be appropriate ingredients to manufacture emulsion sausages. Additionally, non-hydrolyzed silkworm larvae and pupae (*B. mori*) showed noteworthy results for emulsifying properties [33]. Moreover, Alcalase-treated cricket (*G. sigillatus*) proteins were observed to be good emulsifying agents [9].

Hypertension is a major cardiovascular disease, affecting nearly 30% of the adult population of the world [25]. This disease is associated with ACE (EC 3.4.15.1), which is a dipeptidyl-carboxypeptidase that inactivates the vasodilator bradykinin and converts angiotensin-I into angiotensin-II, a powerful vasoconstrictor. Therefore, inhibiting ACE is among the major therapeutic methods for treating hypertension [34]. Overall, in this study, ACE inhibition significantly increased with enzymatic hydrolysis, especially with the Alcalase-treated hydrolysates. Hall et al. [35] indicated that the extensive enzymatic hydrolysis of cricket (*G. sigillatus*) protein enhanced ACE inhibitory activity with IC_50_ values of 0.015–0.040 mg/mL. IC_50_ values of Alcalase hydrolysates in this study was 0.047 mg/mL, which was consistent with the results of a previous study [35]. Interestingly, previous studies have identified the peptides responsible for ACE inhibition from mealworm larvae (*T. molitor*) and silkworm pupae (*B. mori*) hydrolysates [25,26,36]. In this study, the hydrolysate of cricket proteins showed the highest ACE inhibitory activity, and further study will be needed to elucidate the active peptide(s) in this process.

Diabetes mellitus is a chronic disease affecting the health and quality of life of many people worldwide [37]. Previous studies have established that silkworm powder does have some beneficial effects on diabetic patients and this effect was attributed to the mulberry leaves on which silkworms are fed [38]. The hypoglycemic effects of the silkworm are thought to originate from the *α*-glucosidase inhibitory activity of 1-deoxynojirimycin (DNJ), and not proteins or peptides [39]. That said, few studies have investigated the *α*-glucosidase inhibitory activity of peptides from insects. Therefore, in this study, an in vitro α-glucosidase assay was conducted to determine the inhibitory activity of protein hydrolysates from edible insects. Alcalase-treated silkworm pupae and the Flavourzyme and Alcalase mixture-treated mealworm larvae were indicated as the most effective α-glucosidase inhibitors among the protein hydrolysates. However, the hydrolysates inhibited *α*-glucosidase activity by 33.5%–35.0% at 2.0 mg/mL protein concentration, which is too weak an inhibitory activity compared to the acarbose as a positive control (IC_50_: 120 µg/mL, data not shown). The lack of studies on the in vitro *α*-glucosidase inhibitory effects of insect proteins limits the discussion of our results. However, a quantitative structure–activity relationship (QSAR) approach and in silico digestion of proteins from silkworm pupae was performed in order to predict information of regarding the peptides that could have α-glucosidase inhibitory activity [18]. Based on the QSAR, four peptides that had the greatest inhibitory effects were selected and assayed. Among these four peptides, Ser-Gln-Ser-Pro-Ala was identified to be the most potent peptide with an IC_50_ value of 20 µM (~178 µg/mL) equivalent to the activity of acarbose. The anti-diabetic activity of peptides from other species has not been established and further research is necessary to fully elucidate their role in combatting diabetes.

Lastly, we investigated whether the insect protein hydrolysates had anti-inflammatory effects, as depicted by a reduction in NO production from RAW 264.7 cells. Only silkworm pupae protein hydrolysates reduced NO production. Several proteins within the hemocyte and hemolymph from silkworm pupae were confirmed to exert anti-inflammatory activity by reducing NO production in an LPS-stimulated macrophage model [19,40]. Silkworm hemolymph has previously been shown to inhibit inflammation by decreasing NO production, the expression of inducible nitric oxide synthase (iNOS), and the production of pro-inflammatory cytokines [19]. However, the enzymatic hydrolysates used in this study showed decreased anti-inflammatory effects, which was the opposite of what was expected. This might be due to the degradation of functional proteins responsible for the anti-inflammation effects, such as the LPS-binding protein [41], silkworm storage protein 1 [42,43], and immune regulating peptide against infection [44]. These bioactive compounds could be degraded and inactivated during enzymatic hydrolysis.

## 5. Conclusions

We evaluated and compared the physicochemical and physiological properties of hydrolysates from mealworm larvae, crickets, and silkworm pupae. Flavourzyme and Alcalase was used to produce hydrolysates because they showed the highest hydrolytic effects among the four enzymes tested. In addition, the combination of Flavourzyme and Alcalase was also used in order to increase the hydrolytic effects. Generally, the solubility of the hydrolyzed proteins was higher than that of the unhydrolyzed proteins, with mealworm larvae showing the most dramatic increase in solubility at 30%–50%. In terms of emulsifying properties, cricket hydrolysates treated with Alcalase were determined to be the best emulsifiers of the hydrolysates tested due to their emulsifying stability. When insect proteins were treated excessively with Flavourzyme and Alcalase, the emulsifying properties of the hydrolysates decreased. The hydrolysis of the insect proteins also increased the ACE and α-glucosidase inhibitory activity for the three species tested. Among the hydrolysates, the Alcalase-treated cricket proteins showed the greatest inhibition of ACE. With respect to the inhibition of α-glucosidase, Alcalase-treated silkworm pupae and the mixture of Flavourzyme and Alcalase-treated mealworms were determined to be the most effective inhibitors. Lastly, in our investigation into the anti-inflammatory effects of the insect proteins, only silkworm pupae showed anti-inflammatory activity, and this decreased with hydrolysis. Although further research is required, the current examination presented here may help advance the development and transition of insect proteins into a future food and nutraceutical.

## Abbreviations

IC_50_, the half maximal inhibitory concentration; Leu, leucine; Ile, isoleucine; Lys, lysine; Met, methionine; Thr, threonine; Trp, tryptophan; Phe, phenylalanine; Val, valine; Ser, serine; Gln, glutamine; Pro, proline; Ala, alanine; SDS, Sodium dodecyl sulfate; HEPES, 4-(2-Hydroxyethyl)-1-piperazine ethanesulfonic acid; MTT, 3-(4,5-dimethylthiazol-2-yl)-2,5-diphenyltetrazolium bromide; DMEM, Dulbecco’s modified eagle’s medium; DPBS, Dulbecco’s phosphate buffered saline; FBS, fetal bovine serum; DMSO, dimethyl sulfoxide; LPS, lipopolysaccharide; NO, nitric oxide

## References

[1] Cabra V., Arreguin R., Farrés A. (2008). Emulsifying properties of proteins. Bol. Soc. Quim. Mex..

[2] Panyam D., Kilara A. (1996). Enhancing the functionality of food proteins by enzymatic modification. Trends Food Sci. Technol..

[3] Nongonierma A.B., FitzGerald R.J. (2017). Strategies for the discovery and identification of food protein-derived biologically active peptides. Trends Food Sci. Technol..

[4] FitzGerald R., O’Cuinn G. (2006). Enzymatic debittering of food protein hydrolysates. Biotechnol. Adv..

[5] Van Huis A., Van Itterbeeck J., Klunder H., Mertens E., Halloran A., Muir G., Vantomme P. (2013). Edible Insects: Future Prospects for Food and Feed Security.

[6] Kim H.-W., Setyabrata D., Lee Y.J., Jones O.G., Kim Y.H.B. (2016). Pre-treated mealworm larvae and silkworm pupae as a novel protein ingredient in emulsion sausages. Innov. Food Sci. Emerg..

[7] Gould J., Wolf B. (2018). Interfacial and emulsifying properties of mealworm protein at the oil/water interface. Food Hydrocolloid..

[8] Vercruysse L., Van Camp J., Smagghe G. (2005). ACE inhibitory peptides derived from enzymatic hydrolysates of animal muscle protein: A review. J. Agric. Food Chem..

[9] Hall F.G., Jones O.G., O’Haire M.E., Liceaga A.M. (2017). Functional properties of tropical banded cricket (*Gryllodes sigillatus*) protein hydrolysates. Food Chem..

[10] Govorushko S. (2019). Global status of insects as food and feed source: A review. Trends Food Sci. Technol..

[11] Kim S.-K., Weaver C.M., Choi M.-K. (2017). Proximate composition and mineral content of five edible insects consumed in Korea. CyTA J. Food.

[12] Sogari G., Menozzi D., Mora C. (2017). Exploring young foodies’ knowledge and attitude regarding entomophagy: A qualitative study in Italy. Int. J. Gastron. Food Sci..

[13] House J. (2016). Consumer acceptance of insect-based foods in the Netherlands: Academic and commercial implications. Appetite.

[14] Sosa D.A.T., Fogliano V. (2017). Potential of insect-derived ingredients for food applications. Insect Physiology and Ecology.

[15] Mishyna M., Martinez J.-J.I., Chen J., Benjamin O. (2019). Extraction, characterization and functional properties of soluble proteins from edible grasshopper (*Schistocerca gregaria*) and honey bee (*Apis mellifera*). Food Res. Int..

[16] Mintah B.K., He R., Dabbour M., Xiang J., Agyekum A.A., Ma H. (2019). Techno-functional attribute and antioxidative capacity of edible insect protein preparations and hydrolysates thereof: Effect of multiple mode sonochemical action. Ultrason. Sonochem..

[17] Nongonierma A.B., FitzGerald R.J. (2017). Unlocking the biological potential of proteins from edible insects through enzymatic hydrolysis: A review. Innov. Food Sci. Emerg..

[18] Zhang Y., Wang N., Wang W., Wang J., Zhu Z., Li X. (2016). Molecular mechanisms of novel peptides from silkworm pupae that inhibit α-glucosidase. Peptides.

[19] Chang M.-R., Lee W.H., Rhee W.J., Park T.H., Kim E.J. (2013). Anti-inflammatory effects of silkworm hemolymph on lipopolysaccharide-stimulated macrophages. Korean J. Chem. Eng..

[20] Zielińska E., Baraniak B., Karaś M. (2017). Antioxidant and anti-inflammatory activities of hydrolysates and peptide fractions obtained by enzymatic hydrolysis of selected heat-treated edible insects. Nutrients.

[21] Choi B.D., Wong N.A., Auh J.-H. (2017). Defatting and sonication enhances protein extraction from edible insects. Korean J. Food Sci. Anim. Resour..

[22] Pacheco-Aguilar R., Mazorra-Manzano M.A., Ramírez-Suárez J.C. (2008). Functional properties of fish protein hydrolysates from Pacific whiting (*Merluccius productus*) muscle produced by a commercial protease. Food Chem..

[23] Kwon D., Kim G.D., Kang W., Park J.-E., Kim S.H., Choe E., Kim J.-I., Auh J.-H. (2014). Pinoresinol diglucoside is screened as a putative α-glucosidase inhibiting compound in *Actinidia arguta* leaves. J. Korean Soc. Appl. Biol. Chem..

[24] Chang B.W., Chen R.L., Huang I.J., Chang H.C. (2001). Assays for angiotensin converting enzyme inhibitory activity. Anal. Biochem..

[25] Dai C., Ma H., Luo L., Yin X. (2013). Angiotensin I-converting enzyme (ACE) inhibitory peptide derived from *Tenebrio molitor* (L.) larva protein hydrolysate. Eur. Food Res. Technol..

[26] Jia J., Wu Q., Yan H., Gui Z. (2015). Purification and molecular docking study of a novel angiotensin-I converting enzyme (ACE) inhibitory peptide from alcalase hydrolysate of ultrasonic-pretreated silkworm pupa (*Bombyx mori*) protein. Process. Biochem..

[27] Kim S.-K., Jo Y.-Y., Lee K.-G., Kim H.-B., Kim Y.S., Ju W.-T., Jung D.-E., Kweon H. (2015). Modification of the characteristics of silkworm powder by treatment with alkaline protease. Int. J. Ind. Entomol..

[28] De Castro R.J.S., Bagagli M.P., Sato H.H. (2015). Improving the functional properties of milk proteins: Focus on the specificities of proteolytic enzymes. Curr. Opin. Food Sci..

[29] Villamil O., Váquiro H., Solanilla J.F. (2017). Fish viscera protein hydrolysates: Production, potential applications and functional and bioactive properties. Food Chem..

[30] Tavano O.L. (2013). Protein hydrolysis using proteases: An important tool for food biotechnology. J. Mol. Catal. B Enzym..

[31] Yi L., Van Boekel M., Lakemond C. (2017). Extracting *Tenebrio molitor* protein while preventing browning: Effect of pH and NaCl on protein yield. J. Insects Food Feed.

[32] Jung S., Murphy P.A., Johnson L.A. (2005). Physicochemical and functional properties of soy protein substrates modified by low levels of protease hydrolysis. J. Food Sci..

[33] Omotoso O.T. (2015). An evaluation of the nutrients and some anti-nutrients in silkworm, *Bombyx mori* L. (Bombycidae: Lepidoptera). Jordan J. Biol. Sci..

[34] Li-Chan E.C. (2015). Bioactive peptides and protein hydrolysates: Research trends and challenges for application as nutraceuticals and functional food ingredients. Curr. Opin. Food Sci..

[35] Hall F., Johnson P.E., Liceaga A. (2018). Effect of enzymatic hydrolysis on bioactive properties and allergenicity of cricket (*Gryllodes sigillatus*) protein. Food Chem..

[36] Tao M., Wang C., Liao D., Liu H., Zhao Z., Zhao Z. (2017). Purification, modification and inhibition mechanism of angiotensin I-converting enzyme inhibitory peptide from silkworm pupa (*Bombyx mori*) protein hydrolysate. Process. Biochem..

[37] Ryu K.-S., Lee H.-S., Kim K.-Y., Kim M.-J., Sung G.-B., Ji S.-D., Kang P.-D. (2013). 1-Deoxynojirimycin content and blood glucose-lowering effect of silkworm (*Bombyx mori*) extract powder. Int. J. Ind. Entomol..

[38] Ryu K.S. (1997). An activity of lowering blood-glucose levels according to preparative conditions of silkworm powder. Korean J. Sericult. Sci..

[39] Tomotake H., Katagiri M., Yamato M. (2010). Silkworm *(Bombyx mori*) are new sources of high quality protein and lipid. J. Nutr. Sci. Vitaminol..

[40] Kim Y.I., Choi K.H., Kim S.R., Goo T.W., Park S.W. (2017). *Bombyx mori* hemocyte extract has anti-inflammatory effects on human phorbol myristate acetate-differentiated THP-1 cells via TLR4-mediated suppression of the NF-κB signaling pathway. Mol. Med. Rep..

[41] Koizumi N., Morozumi A., Imamura M., Tanaka E., Iwahana H., Sato R. (1997). Lipopolysaccharide-binding proteins and their involvement in the bacterial clearance from the hemolymph of the silkworm *Bombyx mori*. Eur. J. Biochem..

[42] Cha Y.J., Baik J.E., Rhee W.J. (2018). Inhibition of Endoplasmic Reticulum Stress-induced Apoptosis by Silkworm Storage Protein 1. Biotechnol. Bioprocess E.

[43] Zhang K., Kaufman R.J. (2008). From endoplasmic-reticulum stress to the inflammatory response. Nature.

[44] Ishii K., Hamamoto H., Kamimura M., Nakamura Y., Noda H., Imamura K., Mita K., Sekimizu K. (2010). Insect cytokine paralytic peptide (PP) induces cellular and humoral immune responses in the silkworm *Bombyx mori*. J. Biol. Chem..

